# Rotavirus Vaccines at 20 Years: Success, Challenges, and the Road Ahead

**DOI:** 10.3390/vaccines14090815

**Published:** 2026-09-16

**Authors:** Alice S. Carter, Duncan Steele, Mathuram Santosham, Michelle J. Groome, Denise O. Garrett

**Affiliations:** 1 Vaccine Epidemiology, Global Immunization, Sabin Vaccine Institute, Washington, DC 20006, USA; 2 Enterics, Diagnostics, Genomics & Epidemiology, Gates Foundation, Seattle, WA 98109, USA; duncan.steele@gatesfoundation.org; 3 Department of International Health and Pediatrics, Johns Hopkins University, Baltimore, MD 21205, USA; 4 South African Medical Research Council Vaccines and Infectious Diseases Analytics Research Unit, Faculty of Health Sciences, University of the Witwatersrand, Johannesburg 2193, South Africa; michelle.groome@wits-vida.org

**Keywords:** rotavirus, gastroenteritis, diarrhea, enteric

## Abstract

Twenty years after the introduction of rotavirus vaccines, the 15th International Rotavirus Symposium convened researchers, clinicians, immunization program managers, and policymakers to review advances in rotavirus epidemiology, immunology, vaccine development, and implementation. Rotavirus vaccination has contributed to sustained reductions in severe rotavirus hospitalizations and child mortality, yet population-level benefits remain heterogeneous and are constrained by gaps in coverage, supply-related program disruptions, and context-specific vaccine performance. Presenters highlighted evidence from multi-pathogen surveillance confirming that rotavirus remains a leading cause of pediatric diarrhea requiring hospitalization despite widespread vaccine introduction. Accumulating evidence implicates maternally derived antibodies, environmental enteric dysfunction, and early-life microbiome development as contributors to reduced oral vaccine performance in low- and middle-income communities, while recent studies of next-generation vaccine candidates underscore the need for improved correlates of protection and clinically meaningful efficacy endpoints. Collectively, the symposium emphasized that further reductions in rotavirus morbidity and mortality will require strengthening delivery of existing vaccines—including improving coverage, minimizing supply interruptions, and reaching zero-dose and under-immunized children—while sustaining surveillance and investing in integrated enteric disease prevention and next-generation vaccines to optimize protection in high-burden settings.

## 1. Introduction

Since their first introductions two decades ago, rotavirus vaccines have substantially reduced the burden of severe childhood diarrhea, and multiple World Health Organization (WHO)-prequalified vaccines now support routine immunization in more than 140 countries [1,2]. Despite this success, diarrheal disease remains a leading cause of mortality in children younger than five years of age, and rotavirus remains a major cause of severe gastroenteritis in settings where vaccine introduction is delayed, coverage is fragile, and oral vaccine performance is least robust [3,4].

To discuss recent rotavirus research and outline remaining priorities, experts convened in Cape Town, South Africa, for the 15th International Rotavirus Symposium from 30 September to 2 October 2025, hosted by the South African Medical Research Council and the Sabin Vaccine Institute. The symposium, held at the twentieth anniversary of the first national introduction of rotavirus vaccine in Mexico in 2006, brought together more than 250 researchers, vaccine developers, immunization program managers, and policymakers to review the latest scientific advances and identify priorities for the next decade of rotavirus prevention. The scientific community also honored the many scientists who have played a crucial role in rotavirus research, vaccine development and vaccine introduction, including those who are no longer with us and those who continue to advance the field.

Across sessions spanning disease burden, genomics, vaccine impact, determinants of performance, vaccine pipelines, economics, and outbreak response, a shared conclusion emerged: vaccine delivery and innovation must proceed together. Immunization programs must increase and sustain coverage with current vaccines while funders support targeted investments in next-generation vaccines and innovative interventions. This report summarizes key scientific themes and new findings highlighted during the symposium, with an emphasis on evidence relevant to policy and research priorities.

## 2. Conference Sections

### 2.1. Global Success with Persistent Inequities

Rotavirus vaccination is an effective intervention for prevention of severe rotavirus gastroenteritis. Four WHO-prequalified vaccines (RotaTeq [3 dose schedule], Rotarix [2 dose schedule], Rotavac [3 dose schedule], and ROTASIIL [3 dose schedule]) are now in use, and more than 140 countries have introduced rotavirus vaccines into their immunization programs. All rotavirus vaccines are recommended for administration beginning at 6 weeks of age with Diphtheria, Tetanus, and Pertussis vaccine (DTP) and are considered interchangeable if necessary [5].

Recent effectiveness evaluations demonstrate protection against rotavirus gastroenteritis, with higher protection observed against hospitalization for severe rotavirus gastroenteritis (Table 1). Observed vaccine effectiveness is highest during infancy and tends to decline in the second year of life.

Analyses of disease burden have associated vaccine roll-out with large reductions in rotavirus diarrhea. Regional syntheses in sub-Saharan Africa report declines in rotavirus-positive hospitalizations following vaccine introduction [6]. Data presented at the 15th International Rotavirus Symposium spanning 2010–2023 in East and Southern Africa reported large declines in rotavirus positivity following vaccine introduction: in a group of seven countries that introduced vaccine in 2014–2015 (Eritrea, Kenya, Madagascar, Mauritius, Eswatini, Zimbabwe, Mozambique), average rotavirus positivity decreased from 40.5% pre-introduction to 16.6% post-introduction (a 59% reduction). In Lesotho, Seychelles, and Uganda (introduced in 2017), rotavirus positivity declined from an average of 43% before introduction to 18.3% post-introduction (a 57% reduction) [7]. Consistent findings have also emerged from West Africa. In Nigeria, rotavirus positivity among hospitalized children under 5 years declined from 35% before vaccine introduction to 19% after introduction, representing a 45% (95% CI: 37–52) reduction. The largest reductions were observed among infants younger than 12 months, including a 59% adjusted reduction (95% CI: 42–71) in rotavirus positivity among diarrhea hospitalizations presenting with severe dehydration or resulting in death following vaccine introduction [8].

In South Asia, rotavirus vaccines have also demonstrated impact. In India for instance, where the two Indian vaccines were rolled out nationally in 2019, rotavirus-positivity among acute watery diarrhea samples declined from 37% pre-vaccine to 17.5% following vaccine introduction [9]. National evaluations from Mexico, Brazil, Panama, Malawi, and other early-adopting countries have demonstrated reductions in diarrhea-associated mortality following rotavirus vaccine introduction, while subsequent multi-country analyses have estimated substantial declines in global rotavirus mortality attributable to vaccination [10].

**Table 1:** Vaccine effectiveness (VE) estimates from presented studies.

Study Location	Study Population	Doses	Case Definition	Effectiveness Estimate	Notes
Mukuru, Kenya [11]	Children <5 years presenting to outpatient health facilities with acute diarrhea (≥3 loose stools/24 h)	Any	Laboratory-confirmed rotavirus infection (polymerase chain reaction from stool sample)	57.6% (95% CI: 18.1–99.8)	Increased age at disease onset to 12–23 months; genotyping suggests shifting strain distributions alongside sustained burden reductions
Kinshasa, DRC [12]	Children 6–30 months hospitalized for acute watery diarrhea (≥3 loose stools/24 h)	Full series	Laboratory-confirmed rotavirus infection (enzyme immunoassay from stool sample)	60% (95% CI: 20–80%)	
Uganda [13]	Children 4–59 months hospitalized for acute watery diarrhea (≥3 loose stools/24 h)	Full series	Laboratory-confirmed rotavirus infection (enzyme immunoassay from stool sample)	29% (95% CI: −37–63) overall; 62% (95% CI: 9–84) in infants 4–11 months, −69% (95% CI: −401–43) in 12–59 months	
Pakistan [14]	Children 22 weeks–23 months presenting to clinic with diarrhea (≥3 loose stools/24 h) and receiving intravenous hydration	Full series	Laboratory-confirmed rotavirus infection (enzyme immunoassay from stool sample)	31% (95% CI: 13–45) overall; 33% (95% CI: 12–49) in infants 22 weeks–11 months, 24% (95% CI: −20–52) in 12–23 months	VE not calculated for children 24–59 months due to small case numbers
Indonesia [15]	Children <1 year hospitalized with acute gastroenteritis	Full series	Laboratory-confirmed rotavirus infection (enzyme immunoassay from stool sample)	74% (95% CI: 32–90) for full 3-dose vaccination	

### 2.2. Surveillance

As vaccine coverage increases, diarrhea surveillance becomes increasingly important for understanding (i) the residual burden attributable to rotavirus, (ii) the changing contribution of other enteric pathogens to severe disease, and (iii) determinants of severity and mortality among hospitalized children.

The Global Pediatric Diarrhea Surveillance (GPDS) network uses quantitative molecular diagnostics for multiple entero-pathogens across 28 countries. Data presented for 2017–2023 provide evidence that rotavirus remains an important cause of diarrhea requiring hospitalization, while Shigella, norovirus and adenovirus contribute substantially and vary by age and geography [16]. GPDS findings also indicate that mortality risk among hospitalized children is shaped by both host factors and care processes, reinforcing that reductions in mortality will require prevention, increased access to oral rehydration salts, and high-quality clinical management [17].

The Vaccine Impact on Diarrhea in Africa (VIDA) study assessed whether the relationship between moderate-to-severe diarrhea and subsequent growth faltering changed following rotavirus vaccine introduction. Children enrolled in the pre-vaccine Global Enteric Multicenter Study were compared with children enrolled in VIDA after vaccine introduction in the Gambia, Kenya, and Mali, with stunting assessed 2–3 months after an episode of moderate-to-severe diarrhea. Despite reductions in rotavirus burden following vaccine introduction, the association between moderate-to-severe diarrhea and stunting did not change, suggesting reducing diarrhea-associated growth faltering requires integrated approaches targeting multiple enteric pathogens [18].

Genomic surveillance is an increasingly important component of decision-relevant monitoring, and whole-genome sequencing can characterize lineage diversity, detect viral reassortment events, and support interpretation of temporal shifts in circulating strains. Post rotavirus vaccine introduction, substantial genotype diversity has persisted, and available surveillance data have not demonstrated vaccine escape. For example, in South Africa, characterization of DS-1-like G8P[4] strains circulating between 2009 and 2021 identified endemic sub-lineages and evidence of epitope change, illustrating the value of longitudinal sequencing of strains for interpreting viral evolution in the post-vaccine era [19]. Antigenic variability analyses comparing circulating strains with the Rotarix vaccine strain documented amino-acid differences in VP4 and VP7 neutralization epitope regions [19]. Surveillance updates from Kenya identified predominance of G2P[4] alongside detection of G12P[6], G2P[8], and equine-like G3P[8] reassortants, patterns aligned with broader African surveillance reports documenting genotype shifts without loss of vaccine effectiveness [6,11].

Despite rotavirus vaccine introductions, mortality from diarrheal disease remains substantial and is concentrated in settings where vaccine coverage is lower, vaccine timeliness is poorer, and children face higher baseline risks of severe outcomes from diarrheal infections [4,10]. For example, in Soweto, South Africa, acute gastroenteritis accounted for ~13% of analyzed under-5 deaths (12% in infants; 14% in older children), and among acute gastroenteritis deaths, 20% (11 of 55) had rotavirus detected, even in a setting of >90% rotavirus vaccine coverage [20,21]. Observed age shifts and declining second-year protection may reflect waning immunity, intense force of infection, vaccination timeliness and completion, and interactions with maternal antibodies and infant gut ecology [22,23,24,25].

Prof. George Armah of the University of Ghana delivered the Roger Glass plenary lecture on rotavirus surveillance and clinical studies conducted in sub-Saharan Africa. He emphasized the pivotal role of African investigators and trial sites in the development, evaluation, and continued optimization of currently licensed and next-generation rotavirus vaccines. For example, the African Rotavirus Surveillance Network, established with seed funding from WHO in 1998, has continued to document the burden of rotavirus disease in Africa and the impact of the vaccine following introduction. Rotavirus vaccine research in South Africa, Ghana, Mali, Malawi, and Kenya helped build capacity to conduct large clinical trials. Recent efficacy trials on the continent highlight the need for continued development of next generation vaccines with improved efficacy in high-mortality settings. Challenges of the differential efficacy in Africa should serve as a roadmap for future research directions.

### 2.3. Determinants of Variable Oral Vaccine Effectiveness

Oral rotavirus vaccines have lower immunogenicity and effectiveness in high-mortality settings compared to low-mortality settings. Across cohorts, higher maternally derived antibody concentrations, features of enteric dysfunction and inflammation, concurrent infections, and differences in early-life microbiome development have been associated with reduced immune responses to oral vaccines, including rotavirus vaccines [24,25,26].

Maternal antibodies protect babies from rotavirus infection but can contribute to reduced vaccine immunogenicity in early life. Mouse models have shown that reduced vaccine immunogenicity in the presence of maternal antibodies is not due to epitope masking or B-cell receptor co-ligation, but instead appears to be due to maternal antibody-mediated clearance of vaccine virus [27]. These findings place maternal antibody biology at the center of the performance gap and frame a clear translational hypothesis: vaccine strategies that reduce the dependence on gut replication, or that target immune priming when maternal antibodies are lower, could improve protection in high-burden settings.

Complementing mechanistic work, schedule optimization was highlighted as an actionable, near-term strategy in settings with high force of infection. Evidence from a Malawian observational study helped define when risk of disease is highest and maternal interference may be lowest. Infants with the lowest maternal rotavirus-specific IgG levels before vaccination were more likely to respond than those with the highest levels [28]. The same study described population-level kinetics in which rotavirus-specific IgG declined to a nadir at 8.4 months, while severe rotavirus gastroenteritis incidence peaked at around 9 months, suggesting a period of heightened vulnerability when maternally derived protection has waned. The authors proposed that a booster dose between 6 and 8 months could improve durability of protection in settings with high force of infection.

New data indicate potentially modifiable pathways and adjunctive interventions linked to oral vaccine take. Microbiota-derived tryptophan metabolites were reported to inhibit rotavirus infection in experimental systems and associated clinical observations [29,30,31]. In a vaccinated Nicaraguan birth cohort, concentrations of specific human milk oligosaccharides were associated in different directions with oral rotavirus vaccine seroconversion and subsequent rotavirus gastroenteritis risk, including lower risk with higher difucosyllacto-N-hexaose (DFLNH) concentrations and higher risk with higher lacto-N-fucopentaose I (LNFP-I) concentrations [32]. These findings, currently reported in a preprint, illustrate the complex and potentially opposing relationships between HMO composition, vaccine response, and rotavirus susceptibility. A study using activation-induced marker assays in Zambian infants suggested limited induction of circulating VP6-specific T cell responses following oral vaccination, raising questions about tissue localization, timing, antigen selection, and antigen presentation [33,34].

Taken together, these data reinforce a central field challenge: existing immune readouts (including serum IgA) are incomplete predictors of protection. The field is converging on a multi-factor model in which maternal antibodies, mucosal immune development, microbiome, and infection pressure interact.

To synthesize past lessons in rotavirus vaccine immunology, Prof. Harry Greenberg of Stanford University delivered the Ruth Bishop Memorial lecture, journeying through his decades of rotavirus vaccine research, emphasizing the significance of intellectual mentorship and curiosity. As a developer of the reassortant strategies for rotavirus vaccines and scientific advisor to Bharat Biotech’s rotavirus vaccine, he has dedicated much of his research career to elucidating the immune mechanisms of rotavirus infection and vaccination.

### 2.4. The Pipeline of Next-Generation Vaccines

A portfolio of next-generation vaccines is progressing through the development pipeline. Researchers are advancing multiple platforms and antigenic targets, with explicit attention to programmatic fit (including cost, presentation, cold chain, and co-administration), and with decision points grounded in clinically meaningful protection.

Non-oral platforms remain conceptually attractive because they could bypass intestinal constraints that limit performance of live oral vaccines. A parenteral trivalent P2-VP8 subunit candidate demonstrated acceptable safety and elicited robust serum antibody immunogenicity in earlier trials [35]. At the symposium, phase 3 results from Ghana, Malawi, and Zambia showed inferior protection in the first two years of life compared with Rotarix; these findings were subsequently published, with a primary relative vaccine efficacy against severe rotavirus gastroenteritis of −77.7% (95% CI, −162.4 to −21.5) [36].

A preliminary correlates-of-risk analysis from this P2-VP8 vaccine study illustrated the complexity of biomarker interpretation. For example, in the control arm vaccinated with Rotarix, elevated day 85 serum IgA responses were associated with lower severe rotavirus gastroenteritis risk, consistent with previous studies. Meanwhile, in infants vaccinated with P2-VP8, higher post-vaccination anti-VP8 IgG responses to P[4] were associated with increased risk of severe rotavirus gastroenteritis [37]. The mechanisms for this association remain unclear, and better immune endpoints—and ultimately validated correlates of protection—remain high-value goals for accelerating vaccine development and evaluation.

Neonatal immunization strategies are particularly relevant where force of infection is high and where opportunities for infant vaccination after birth are frequently missed. An oral human neonatal rotavirus vaccine RV3-BB, designed for administration from birth, has demonstrated immunogenicity and safety in neonatal and infant schedules in Malawi and Indonesia [38,39].

In parallel, preclinical work continues to explore alternative antigens and delivery approaches, including strategies targeting VP6 with mucosal delivery systems that have shown protection in animal models [40].

### 2.5. Supply, Financing, and Program Quality Shape Impact

In many settings, near-term gains will depend on strengthening program delivery. Consistently achieving high coverage and complete dosing depends on health-system resilience, supply reliability, and sustainable financing, particularly as countries transition from external support and face increasing vaccine and delivery costs.

45 of 54 African countries have introduced rotavirus vaccine. 2024 complete series coverage was around 65%--higher than the global estimate of 59% coverage, but well below the 90% goal, with substantial regional heterogeneity and multiple countries reporting very low coverage [41,42]. Unfortunately, rotavirus vaccine coverage remains lower than DTP coverage in Africa.

Product switches have been common (notably 2019–2023), prompted by global vaccine supply shortages, cold chain constraints, or cost. Experience in the WHO African Region indicates that product switches can be implemented successfully but require coordinated procurement planning, cold-chain assessment, standardized health-worker training materials, clear guidance for interrupted or mixed schedules, and updated recording and reporting tools [43]. Zambia’s Rotarix-to-Rotavac transition illustrated operational challenges that can occur during stock-outs and product changes, including risks of missed opportunities and provider confusion regarding mixed schedules [44].

Crucially, several presentations addressed the realities of delivery in fragile and underserved contexts. Border closure and program interruptions in Palestine from mid-2024 were followed by an increase in diarrheal cases [45]. A community-driven strategy from refugee-hosting districts in Uganda highlighted practical steps to strengthen uptake, including community engagement, trust-building, and service delivery adaptations [46].

Economic evaluations continue to support rotavirus vaccination as a cost-effective intervention in high-burden settings and can inform product selection and long-term domestic financing strategies. Analyses from Egypt and Indonesia showed cost-effectiveness estimates are sensitive to vaccine price, delivery costs, and assumptions about coverage and timeliness; therefore, economic evidence should be interpreted alongside realistic assessments of program capacity, equity, and supply resilience [47,48].

## 3. Discussion

Two decades into the rotavirus vaccine era, the evidence supports substantial and sustained public health benefit, while also delineating persistent scientific and programmatic constraints that limit equity. Rotavirus vaccines can deliver rapid and sustained reductions in severe disease when programs achieve and maintain high, timely coverage. Further reductions in morbidity and mortality will require coordinated action across vaccine development, surveillance, and delivery systems, sustaining high coverage with currently available products, protecting program continuity during transitions, and rigorously evaluating schedule innovations and next-generation candidates using endpoints linked to severe disease in the populations at greatest risk.

### 3.1. Key Scientific Discussions

- In sentinel surveillance, rotavirus remains an important cause of diarrhea requiring hospitalization; mortality risk among hospitalized children is strongly shaped by clinical severity and health-system access.
- Vaccine coverage in high-burden regions remains heterogeneous and vulnerable to disruption, particularly during procurement transitions and stock-outs.
- Maternal antibodies and early-life microbiome development are consistently associated with variability in oral vaccine immunogenicity, providing plausible targets for schedule-based strategies and adjunctive interventions.

### 3.2. Future Research and Implementation Priorities

- Improve timeliness and completion of rotavirus vaccination and reduce missed opportunities in high-burden areas.
- Strengthen supply resilience and switch readiness through coordinated forecasting, communication, and rapid subnational monitoring during transitions.
- Sustain surveillance with multi-pathogen attribution and genomics to support interpretation of residual burden and detect epidemiologic shifts.
- Evaluate feasible schedule modifications (including neonatal and booster strategies) using disease endpoints and implementation measures.
- Advance a diversified portfolio of next-generation candidates with development pathways anchored to clinical protection.

Together, these implications support an integrated agenda: protect existing gains through immunization program quality and supply resilience; invest in surveillance to quantify residual rotavirus burden and to contextualize shifts in other enteric pathogens; and pursue innovation in vaccines and schedules that is evaluated against disease outcomes in the settings where the burden remains highest.

Africa is poised to lead the way, as a principal setting for rotavirus surveillance, clinical evaluation, implementation research, and next-generation vaccine development.

## 4. Conclusions

Twenty years into the rotavirus vaccine era, the 15th International Rotavirus Symposium demonstrated how the field has evolved from establishing vaccine efficacy and supporting global introduction to understanding and addressing the residual burden of rotavirus disease. Continued investigation of alternative vaccination schedules, identification and validation of correlates of protection, and development of next-generation vaccine platforms proceed alongside efforts to strengthen routine immunization systems and reach children at greatest risk. Future progress will require translating advances in surveillance, immunology, and vaccine development into strategies that improve the magnitude and durability of protection in populations where current vaccines perform least well.

## Data Availability

No new data were created or analyzed in this study. Data sharing is not applicable to this article.

## Abbreviations

The following abbreviations are used in this manuscript:

CI	Confidence interval
DTP	Diphtheria, tetanus, pertussis vaccine
GPDS	Global Pediatric Diarrhea Surveillance network
VE	Vaccine effectiveness
VIDA	Vaccine Impact on Diarrhea in Africa study
WHO	World Health Organization
